# Quality of Life Changes during the COVID-19 Pandemic for Caregivers of Children with ADHD and/or ASD

**DOI:** 10.3390/ijerph18073667

**Published:** 2021-04-01

**Authors:** Keith W. Pecor, Georgia Barbayannis, Max Yang, Jacklyn Johnson, Sarah Materasso, Mauricio Borda, Disleidy Garcia, Varsha Garla, Xue Ming

**Affiliations:** 1 Department of Biology, The College of New Jersey, Ewing, NJ 08628, USA; pecor@tcnj.edu; 2 Department of Neurology, Rutgers University-New Jersey Medical School, Newark, NJ 07103, USA; gnb30@scarletmail.rutgers.edu (G.B.); jaj176@njms.rutgers.edu (J.J.); garciadisleidy@gmail.com (D.G.); 3 College of Arts and Sciences, University of Pennsylvania, Philadelphia, PA 19104, USA; maxyang@sas.upenn.edu (M.Y.); sematerasso@gmail.com (S.M.); 4 Department of Neurology, Montefiore Medical Center, Bronx, NY 10467, USA; mborda@montefiore.org; 5 College of Arts and Sciences, Vanderbilt University, Nashville, TN 37212, USA; varsha.garla@vanderbilt.edu

**Keywords:** attention-deficit/hyperactivity disorder, ADHD, autism spectrum disorder, ASD, caregiver, COVID-19, quality of life, QOL, family burden, Pediatric Quality of Life Inventory^TM^

## Abstract

The COVID-19 pandemic has presented many challenges to caregivers of children. Families with children with attention-deficit/hyperactivity disorder (ADHD) and/or autism spectrum disorder (ASD) are an understudied but potentially vulnerable population to changes during the outbreak. As such, the aim of this study was to contrast quality of life for caregivers of children with ADHD and/or ASD, before and during the pandemic, compared to caregivers of neurotypical (NT) children. Total, Parent Health-Related Quality of Life, and Family Functioning Summary Scores from the Family Impact Module of the Pediatric Quality of Life Inventory^TM^ were contrasted among caregivers of children with ADHD, ASD, comorbid ADHD and ASD, and NT development. For all scores, caregivers of ADHD and/or ASD children reported lower quality of life, both before and during the pandemic, in comparison to caregivers of NT children. For all diagnoses, quality of life decreased during the pandemic, but caregivers of children with ADHD and/or ASD reported a greater decrease in quality of life than caregivers for NT children. There are limitations to this study in terms of the composition of the sample and the survey methodology, but we are able to conclude that caregivers of children with ADHD and/or ASD have been disproportionately affected by the pandemic, and it is imperative that these families receive additional resources and support to improve their quality of life.

## 1. Introduction

The COVID-19 pandemic has resulted in numerous adjustments to daily life for children and their caregivers, including stay-at-home orders, remote learning due to school closures, and new social distancing recommendations. Although these measures are in place to assist from a public health perspective, they pose new risks and burdens for children and their caregivers. Children are a particularly vulnerable population during emergencies and natural disasters. Disruptions may occur during critical developmental periods that can lead to increased incidence of post-traumatic stress disorder (PTSD), anxiety disorders, and depression [1,2]. A recent study on the COVID-19 pandemic found that 14% of surveyed parents reported worse physical and behavioral health for their children in June 2020 than in March 2020 [3]. Importantly, these COVID-19-related adjustments have negatively impacted the well-being of the parents and caregivers of these children [4].

While there is potential for distress in all children, these concerns are amplified for those children with neurodevelopmental conditions, such as attention-deficit/hyperactivity disorder (ADHD) and/or autism spectrum disorder (ASD). For example, children with ASD are more prone to anxiety than neurotypical (NT) individuals, which can lead to maladaptive behavior [5]. An emergency can heighten such responses, as was seen during the 2009 earthquake in L’Aquila, Italy, in which children with ASD suffered in the fields of communication, daily living, socialization, and motor skills [6]. During the COVID-19 pandemic, it has been shown that children with ADHD and ASD engaged in more intense and frequent behavioral problems compared to before the pandemic [7,8,9,10,11,12]. Furthermore, individuals with intellectual disability may become preoccupied with pandemic information, leading to increased anxiety and paranoia [13].

In addition to impacts on children, the COVID-19 pandemic and responses to it have also affected parents and other caregivers negatively [3,14], including increased incidence of post-traumatic stress symptoms [15]. School closures alone have been attributed to higher rates of caregiver stress, child abuse, and violence against children [16]. Families with children with ADHD have reported reduced access to mental health and education services and challenges with remote learning [8,17]. Moreover, many families with ASD receive fewer, if any, crucial remote special education services and face disruptions in behavioral, speech, and occupational therapy services during COVID-19 [9,18,19,20,21,22].

Children with ADHD and/or ASD that receive remote special needs services may lose critical information and feedback from school systems and interventional therapists, as well as in-person support. For instance, an instructor for a student with ASD in a classroom may observe certain triggers of maladaptive behaviors that student may carry out after school. These triggers and outcomes that would have otherwise been conveyed to their caregivers in an in-person academic setting may be lost in a virtual classroom. Moreover, the invaluable insight gained from behavioral, speech, and occupational therapists during in-person sessions with children with ASD and/or ADHD may be compromised virtually. Further, the social, emotional, and psychological support parents receive during in-person interactions with school systems or advocacy groups may also suffer during the COVID-19 pandemic [18,21,22]. These factors could result in increased caregiver burden and stress. As with the children themselves, there is the potential for more difficulty when caring for a child with developmental disorders, because it is well-known that children with ASD are resistant to changes in routine [18,22,23]. In times of non-crisis, caregivers of children with ADHD and ASD report higher levels of stress and anxiety compared to parents of NT individuals [24,25,26,27]. Further, families caring for children with ASD have more difficulties obtaining health care and support from agencies than families of children with other special health care needs [28].

Quality of life (QOL) is a multidimensional construct that comprises an individual’s perceived psychological, social, emotional, and physical functioning, and is often used to examine well-being and burden in families with neurodevelopmental disorders [29,30,31,32]. Importantly, poor quality of life in caregivers is associated with higher caregiver stress and burden, more maladaptive behaviors in children, and, ultimately, worse family functioning [33,34,35].

Individual measures of quality of life (e.g., depression, anxiety, stress) in response to the COVID-19 pandemic have been studied, but to our knowledge, few studies have examined the effects of COVID-19 in families with neurodevelopmental disorders from a multifactor, holistic perspective. To address this important gap in research, the goal of this study was to assess quality of life for caregivers of children with ADHD and/or ASD compared to NT children, both before and during the COVID-19 pandemic, and to determine how the pandemic has affected these individuals.

## 2. Materials and Methods

### 2.1. Participants

Participants were caregivers of children who were NT, diagnosed with ADHD, diagnosed with ASD, or diagnosed with comorbid ADHD and ASD. Caregivers self-reported diagnosis with ADHD and/or ASD. All participants had to read and comprehend English and have Internet access in order to complete the web-based questionnaire.

A convenience sample of caregivers was recruited from medical records and from the community. Participants with children seen by physicians at Rutgers New Jersey Medical School or University Hospital were recruited through email, text message, and telephone invitations based on medical records. Members of the general public were recruited through flyers, Rutgers email blasts, social media posts, and advertisements in *New Jersey Family*. Participants gave informed consent, and no identifying information was collected from any individual. Procedures for human subjects were approved by the Institutional Review Board for Rutgers University.

### 2.2. Procedure

Participants completed an electronic questionnaire on Qualtrics or Google Forms that included questions about demography (e.g., caregiver age, ethnicity, gender), family situation (e.g., household members, number of caregivers, employment), child’s diagnosis, and the full complement of questions from the Pediatric Quality of Life Inventory^TM^ (PedsQL^TM^) Family Impact Module (FIM) version 2.0, which is designed to assess the quality of life of parents and families with children with chronic health concerns [29]. This questionnaire was shown to be valid and reliable in families with children with neurological disorders, such as cerebral palsy [29].

The FIM consists of 36 statements in the categories of physical functioning, emotional functioning, social functioning, cognitive functioning, communication, worry, daily activities, and family relationships. The participant rates each statement on a Likert scale of 0 (never a problem), 1 (almost never a problem), 2 (sometimes a problem), 3 (often a problem), or 4 (almost always a problem). For each section of the FIM, participants were asked to rate the statements based on their perceptions before and during the pandemic (pre-COVID-19, COVID-19). The pre-COVID-19 timeframe was defined as before 1 December 2019, and COVID-19 was the time that the survey was taken. A total of 707 questionnaires were submitted. From these, 127 participants did not complete the entire questionnaire, and therefore they were excluded, leaving a final sample of 580 questionnaires. Surveys in the final sample were completed between 29 April and 29 July 2020, but the majority (89.8%) were completed between 15 June and 15 July 2020. Thus, the time spent living with the pandemic was relatively conserved across participants.

### 2.3. Data Analysis

Per the instructions for analyzing the FIM, Likert scores were reverse-transformed to a 100-point scale (0 = 100, 1 = 75, 2 = 50, 3 = 25, and 4 = 0). Thus, a higher transformed score indicates better quality of life. Three aggregate scores for quality of life were calculated for each participant. Total Score was the sum of all answers in the questionnaire divided by 36 (the total number of statements). Parent Health-Related Quality of Life (HRQL) Summary Score was the sum of all answers in the physical, emotional, social, and cognitive functioning sections divided by 20 (the total number of statements in those sections). Finally, Family Functioning Summary Score was the sum of all answers in the daily activities and family relationships sections divided by 8 (the total number of statements in those sections).

The three aggregate scores were analyzed using a separate repeated-measures analysis of variance (ANOVA) for each score, with diagnosis as the between-subjects factor and response time period (pre-COVID-19, COVID-19) as the within-subjects factor. For every measure, there was a significant effect of diagnosis, time, and most importantly, the interaction between diagnosis and time (see Results). Due to the significant interaction, additional analyses were required to determine the relationships among variables. First, one-way ANOVAs were run for each score with diagnosis as the between-subjects factor for pre-COVID-19 responses alone and COVID-19 responses alone. Second, paired *t*-tests were run to contrast pre-COVID-19 and COVID-19 responses within each diagnosis for each score. To avoid increased risk of Type 1 error due to multiple comparisons, we adjusted α to 0.008 using a Bonferroni correction.

In addition, effect sizes in the form of Cohen’s *d* for unequal sample sizes were calculated for each analysis [36]. By convention, these effect sizes are categorized as follows: ≥0.2 (small), ≥0.6 (medium), and ≥0.8 (large) [37]. Effect sizes provide an additional metric for assessing differences among groups and can be useful in studies such as this, in which the response variables are scores without defined units. Statistical calculations were made using a combination of IBM SPSS Statistics v. 25 and Microsoft Excel v. 16.4.

## 3. Results

The participants were largely middle-aged females (Table 1). Of the 575 participants that provided ethnicity and age data, 319 (54%) identified as Caucasian females, and these individuals had a mean age (46.4 years) comparable to the sample as a whole (41.9 years).

For the repeated-measures ANOVAs, there were significant effects of diagnosis, time, and the interaction between diagnosis and time for all three scores (Table 2).

**Figure 1:**
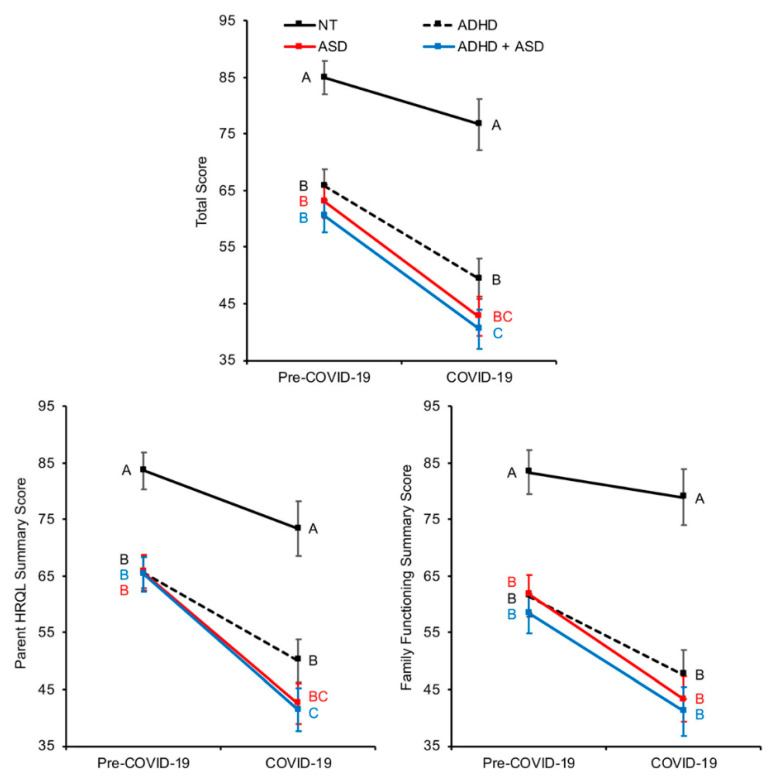
Mean Total Score, Parent HRQL Summary Score, and Family Functioning Summary Score (± 95% confidence intervals) pre-COVID-19 and COVID-19 for caregivers of children identified as NT, ADHD, ASD, and comorbid ADHD and ASD. Different letters represent different statistical groupings (*p* < 0.05) within each time period. With the exception of Family Functioning for caregivers of NT children, all scores for caregivers within each group of children differed significantly between the two time periods (Table 4).

After further analysis with one-way ANOVAs and paired *t*-tests, Total, Parent HRQL Summary, and Family Functioning Summary Scores all showed the same three trends. First, the scores were significantly higher (i.e., greater quality of life) for the caregivers of NT children than the caregivers of individuals diagnosed with ADHD, ASD, or comorbid ADHD and ASD, and effect sizes were medium-to-large, both before and during the pandemic (Table 3, Figure 1).

Before the pandemic, there were no differences among caregivers of children with ADHD, ASD, or comorbid ADHD and ASD for any score. During the pandemic, there were no differences among caregivers of children with ADHD, ASD, or comorbid ADHD and ASD for Family Functioning Summary Score. For Total Score and Parent HRQL Summary Score, caregivers of children with comorbid ADHD and ASD reported significantly lower scores than caregivers of children with ADHD alone. Caregivers of children with ASD alone reported scores that were intermediate and did not differ from either other group. In all cases, the effect sizes were small or lower. This indicates the scores among the groups, despite statistical significance in current Total and Parent HRQL Summary Scores, were not substantially different (Table 3, Figure 1).

Second, quality of life decreased significantly for all caregivers from pre-COVID-19 to COVID-19, with the exception of Family Functioning for caregivers of NT children (Table 4, Figure 1). Third, and most importantly, the magnitude of the decrease in quality of life pre-COVID-19 to COVID-19 was greater for caregivers of children with ADHD, ASD, and comorbid ADHD and ASD than for caregivers of NT children. This can be seen in the higher slope of the lines connecting the markers for pre-COVID-19 and COVID-19 scores (Figure 1) and the smaller effect sizes for the *t*-tests for caregivers of NT children compared to the effect sizes for caregivers of children with ADHD, ASD, and comorbid ADHD/ASD (Table 4).

## 4. Discussion

The goal of the present study was to determine the impact of the COVID-19 pandemic on the quality of life for caregivers of children diagnosed with ADHD and/or ASD in comparison to NT children using a reliable and validated questionnaire. Caregivers of NT children had higher scores (i.e., greater quality of life) for all measures compared to caregivers for children afflicted with ADHD and/or ASD before the COVID-19 pandemic. These findings are consistent with past quality of life studies in families [24,30,38,39,40,41,42,43,44,45,46,47,48]. When considering the effects of the COVID-19 pandemic on quality of life, caregivers in all groups reported decreased quality of life compared to before the pandemic, and caregivers of children with ADHD and/or ASD reported significantly decreased quality of life compared to caregivers for NT children. Among caregivers for children with ADHD and/or ASD, differences in quality of life were minor or absent.

Our findings are consistent with other studies in terms of both NT children and those with neurodevelopmental disorders. As in Spinelli et al., the quality of life for caregivers of NT children was lower during COVID-19 in our sample [4]. Similarly, our results align with a 2020 Stress in America™ poll, in which over 60% of parents surveyed in the general population reported increased stressors related to health care services, remote learning, social distancing, basic needs, and missed milestones during the COVID-19 pandemic [49]. Additional studies have found a negative impact of the COVID-19 pandemic on parents and other caregivers of NT children [3,14]. In populations with neurodevelopmental disorders, our findings align with studies that indicated caregivers of ASD children suffered from increased stress, distress, fear, anxiety, depression, emotional dysregulation, and decreased mood during the COVID-19 period [9,21,50,51,52,53]. Other empirical studies investigating the impact of the COVID-19 pandemic on caregiver quality of life in families with ADHD are limited, though Shah et al. reported that caregivers of ADHD children noted a higher frequency of negative interactions with their children, including increased irritability, verbal abuse, and punishment [54].

It is important to note that poor caregiver well-being can in turn negatively impact a child’s well-being and quality of life [4]. Studies during the COVID-19 pandemic suggest ASD caregiver anxiety levels correlate with the severity of ASD-related behavioral problems in their children [9], and ASD children’s ability to thrive is associated with their caregiver’s coping strategies [12]. Moreover, Ueda et al. found an association between caregiver stress and maladaptive behavior in a cohort of children with ADHD, ASD, and/or specific learning disabilities [35]. As such, we speculate that the lower quality of life reported by caregivers for children with ADHD and/or ASD may result in a greater negative impact by hindering their ability to provide support and resources to their vulnerable children, and ultimately lead to a lower quality of life for their children.

We acknowledge that some children with ASD display an affinity for technology [18] and may potentially benefit from remote learning [55], but both studies and our clinical observations indicate that a substantial portion of caregivers of children with ASD commonly report concern and distress over their children showing or potentially developing developmental, behavioral, and academic regressions due to disruptions to routines, physical distancing measures, and decreased behavioral, educational, and mental health services during the COVID-19 pandemic [12,20,21,22]. In addition, caregivers of children with ADHD frequently describe stressors associated with remote learning related to distractibility, fewer academic accommodations, and at-home confinement [17]. Caregivers of children with ADHD and/or ASD have to invest additional time and effort, many without formal education or training, to ensure their children engage in remote learning and therapy services, potentially contributing to increased burden and worse quality of life [11,12].

Measures to improve the quality of life for families could be implemented, such as organized outdoor camps with proper social distancing to relieve the families’ burden, home visits to help caregivers complete essential daily activities (e.g., grocery shopping, supervising siblings’ remote learning), and/or giving parents respite care. Caregivers of ASD children cite a need for more support during the COVID-19 pandemic [22,53], and providing psychological support for parents with online counseling, wellness checks, and social events could also boost their morale and lessen their burden. For instance, routine communication and telehealth mental health checks between physicians and caregivers of children with chronic disorders may improve the caregiver’s quality of life, as seen in a recent study where 84% of mothers of children with cystic fibrosis reported decreased COVID-19-related anxiety after a telehealth interview with their child’s physician [56].

In addition, caregivers of children with ADHD and/or ASD can learn behavioral strategies and interventions through telehealth training programs to help reduce their burden and improve their well-being [57]. An innovative staged-based telehealth protocol- which includes acceptance and commitment therapy, parent-focused preference assessment, parent-led activity-based instruction, and parent-implemented intervention-has been developed to provide support to families with children afflicted with ASD [58]. Moreover, a 12-week, mobile-based parent training reduced stress, anxiety, and depression in mothers of ASD children during COVID-19 [59], and online support and training programs effectively supported caregivers of children with ASD during COVID-19 [60,61]. Behavioral parent training is a well-established psychosocial treatment with the strongest evidence base showing positive outcomes in children with ADHD and improvements in parenting [62,63,64,65,66]. A preliminary study implementing telepsychology group behavioral parent training for ADHD children during the COVID-19 pandemic resulted in mostly positive outcomes with efficacy, high treatment fidelity, and parental satisfaction comparable to in-person sessions [67], and a pilot study implementing a text-message based intervention with behavioral parent training principles for ADHD proved feasible and led to satisfaction in a proportion of the caregivers [54,66,68]. While remote parent-guided programs and interventions in families with children with ADHD and/or ASD show promising results in potentially improving caregivers’ quality of life [57,63,67,69], these virtual strategies and interventions are still in their infancy and require further exploration [54,57,59,60,67].

The resources for such support mechanisms should be a consideration in government appropriations given that caregivers of children with ADHD and/or ASD face greater financial burdens, which may be exacerbated in the times of COVID-19 due to employment, housing, and food hardships [18,28,70,71,72]. Advocacy groups such as Autism Speaks and Children and Adults with Attention-Deficit/Hyperactivity Disorder have been urging the United States Congress to provide additional financial support to families of children with disabilities, including for the use of home and community-based services, telehealth services, and economic impact payments [73,74]. At the local level, it is imperative for physicians, educators, therapists, health policy workers, and those in the families’ social networks to consider allocating support to affected families during this time. Additional resources are especially important for this particularly vulnerable population, as due to COVID-19, many families have lost critical in-person formal support (e.g., behavioral therapists, speech-language pathologists, and tutors).

This study has some limitations due to both the sample and methodology. First, the participants were a convenience sample not matched by sex, age, ethnicity, and/or socioeconomic status, and there was not enough representation to allow for analysis of these characteristics. From survey questions, we do know that a majority of participants were middle-aged, Caucasian females, but it is unclear how representative this group is broadly. It is reasonable to assume that the majority of participants were caregivers from New Jersey because most recruitment sources were largely based in New Jersey. The sample selection would be comparable to other convenience samples of online survey sources.

Second, the methodology restricted participation and may have impacted the results. Only caregivers who had Internet access and read and understood English were able to participate. Further, diagnosis was self-reported and did not include any measure of the degree of disability. Moreover, anonymous participation precluded identification of participant recruitment sources (i.e., hospital, clinic, or general public). Finally, recall bias [75] may have influenced caregiver responses to their quality of life as they may have inaccurately reported details of their family’s pre-COVID conditions.

We recommend that future studies build on our work by explicitly considering demographics and diagnosis in both the study design and participant recruitment. Healthcare access is not uniform among individuals from different demographic groups [76], and Black and Hispanic Americans have been disproportionately affected by COVID-19 [49,77,78,79,80,81]. Further, given the wide range of cognitive and behavioral presentations in ADHD and ASD, future studies should include questions about the child’s diagnosis beyond simply “yes” or “no” [82].

## 5. Conclusions

This study showed that quality of life as measured by the PedsQL^TM^ FIM version 2.0 was lower for caregivers of children with ADHD and/or ASD than for caregivers of NT children before and during the COVID-19 pandemic. Further, quality of life declined for all caregivers during the pandemic, but did so more dramatically for caregivers of children with neurological conditions in this cohort. Future studies can build on this work by examining quality of life when additional factors, such as socioeconomics, ethnicity, and degree of impairment, are more explicitly considered in families with children with neurodevelopmental disorders.

## Figures and Tables

**Table 1:** Demographic characteristics of caregivers.

	Diagnosis			
	NT	ADHD	ASD	ADHD + ASD
Sample Size	91	152	170	167
Age (years)				
N ^1^	90	150	170	165
Mean	39.6	43.3	50.3	45.3
Range	20–59	16–79	26–69	23–66
Ethnicity (%)				
African-American	5.5	7.9	10.0	4.2
Asian	45.1	4.6	13.5	9.6
Caucasian	40.7	68.4	54.1	62.9
Hispanic	4.4	12.5	15.3	16.2
More than one choice or prefer not to say	4.4	6.6	7.1	7.2
Gender (%)				
Female	87.9	92.1	91	92.2
Male	9.9	7.9	9	7.2
Prefer not to say	2.2	0	0	0.6

NT-neurotypical; ADHD-attention-deficit/hyperactivity disorder; ASD-autism spectrum disorder. ^1^ Age was free-response, and some participants responded with an answer other than a number, e.g., “Forties.” These responses were excluded from the means calculations.

**Table 2:** Results from the repeated-measures ANOVAs.

		Total Score		Parent HRQL Summary Score		Family Functioning Summary Score	
	df	*F*	*p*	*F*	*p*	*F*	*p*
Diagnosis	3,576	59.8	<0.001	39.84	<0.001	43.9	<0.001
Time	1,576	428.44	<0.001	453.75	<0.001	208.38	<0.001
Diagnosis × Time	3,576	10.28	<0.001	9.7	<0.001	9.32	<0.001

**Table 3:** Results from the one-way ANOVAs and effect size (Cohen’s *d*) calculations.

	Total Score		Parent HRQL Summary Score		Family Functioning Summary Score	
pre-COVID19	*F*	*p*	*F*	*p*	*F*	*p*
	39.42	<0.001	21.94	<0.001	27.29	<0.001
Effect Sizes						
NT v ADHD	1.12		1.06		1.02	
NT v ASD	1.23		0.95		1.01	
NT v ADHD + ASD	1.43		0.99		1.14	
ADHD v ASD	0.14		0.00		0.01	
ADHD v ADHD + ASD	0.29		0.01		0.13	
ASD v ADHD + ASD	0.14		0.01		0.15	
COVID-19	*F*	*p*	*F*	*p*	*F*	*p*
	56.81	<0.001	39.42	<0.001	44.09	<0.001
Effect Sizes						
NT v ADHD	1.23		0.99		1.20	
NT v ASD	1.49		1.32		1.37	
NT v ADHD + ASD	1.61		1.30		1.38	
ADHD v ASD	0.29		0.32		0.16	
ADHD v ADHD + ASD	0.39		0.36		0.23	
ASD v ADHD + ASD	0.10		0.04		0.08	

NT-neurotypical; ADHD-attention-deficit/hyperactivity disorder; ASD-autism spectrum disorder.

**Table 4:** Results from the paired *t*-tests and effect size (Cohen’s *d*) calculations before and during COVID-19 for each diagnosis.

		Total Score		Parent HRQL Summary Score		Family Functioning Summary Score	
	df	*t*	*p*	*t*	*p*	*t*	*p*
NT	90	5.21	<0.001	5.70	<0.001	2.40	0.02
ADHD	151	11.76	<0.001	11.94	<0.001	8.03	<0.001
ASD	169	13.89	<0.001	13.93	<0.001	10.54	<0.001
ADHD+ASD	166	12.95	<0.001	13.48	<0.001	9.60	<0.001
Effect Sizes							
NT		0.45		0.51		0.20	
ADHD		0.79		0.75		0.55	
ASD		0.95		1.05		0.74	
ADHD + ASD		0.96		1.06		0.65	

NT-neurotypical; ADHD-attention-deficit/hyperactivity disorder; ASD-autism spectrum disorder.

## Data Availability

Data can be requested from the corresponding author.
